# Leaf Color Classification and Expression Analysis of Photosynthesis-Related Genes in Inbred Lines of Chinese Cabbage Displaying Minor Variations in dark-green Leaves

**DOI:** 10.3390/plants12112124

**Published:** 2023-05-27

**Authors:** Xiangjie Su, Xiaonan Yue, Mingyu Kong, Ziwei Xie, Jinghui Yan, Wei Ma, Yanhua Wang, Jianjun Zhao, Xiaomeng Zhang, Mengyang Liu

**Affiliations:** State Key Laboratory of North China Crop Improvement and Regulation, Key Laboratory of Vegetable Germplasm Innovation and Utilization of Hebei, Collaborative Innovation Center of Vegetable Industry in Hebei, College of Horticulture, Hebei Agricultural University, Baoding 071000, China

**Keywords:** slight differences in leaf color, leaf color classification, photosynthesis-related genes, relative expression analysis

## Abstract

The leaves of the Chinese cabbage which is most widely consumed come in a wide variety of colors. Leaves that are dark green can promote photosynthesis, effectively improving crop yield, and therefore hold important application and cultivation value. In this study, we selected nine inbred lines of Chinese cabbage displaying slight differences in leaf color, and graded the leaf color using the reflectance spectra. We clarified the differences in gene sequences and the protein structure of ferrochelatase 2 (*BrFC2*) among the nine inbred lines, and used qRT-PCR to analyze the expression differences of photosynthesis-related genes in inbred lines with minor variations in dark-green leaves. We found expression differences among the inbred lines of Chinese cabbage in photosynthesis-related genes involved in the porphyrin and chlorophyll metabolism, as well as in photosynthesis and photosynthesis-antenna protein pathway. Chlorophyll b content was significantly positively correlated with the expression of *PsbQ*, *LHCA1_1* and *LHCB6_1*, while chlorophyll a content was significantly negatively correlated with the expression *PsbQ*, *LHCA1_1* and *LHCA1_2*. Our results provide an empirical basis for the precise identification of candidate genes and a better understanding of the molecular mechanisms responsible for the production of dark-green leaves in Chinese cabbage.

## 1. Introduction

Chinese cabbage (*Brassica rapa* L. ssp. *pekinensis*) belongs to the genus Brassica of the family Cruciferae. An economically important crop, Chinese cabbage is the most extensively cultivated and most consumed leafy vegetable in China. In recent years, consumers have placed additional demands on the physical characteristics of Chinese cabbage varieties, and in particular characteristics relating to leaf color. For instance, Chinese cabbage varieties with dark-green leaves are more likely to be favored by consumers. Chinese cabbage varieties with dark-green leaves have a higher economic value because dark-green leaves can promote photosynthesis, effectively improving yield and quality. Such varieties also provide ideal material for research on the structure of photosynthetic systems and gene regulation mechanisms.

The color of a plant leaf, usually expressed as green, is a comprehensive expression of various pigments in the chloroplast. In higher plants, the biosynthesis of chlorophyll roughly occurs in three steps. The first involves the formation of δ-aminolevulinic acid (ALA); this is a rate-limiting step in the biosynthesis of chlorophyll. The second step is the synthesis from ALA to Proto IX, which reacts with Mg chelatase to synthesize chlorophyll, and with ferrochelatase to synthesize heme. In the final step, chlorophyll a and chlorophyll b are synthetized through a series of reactions. The biosynthesis of the chlorophyll process involves 19 steps of reaction and 15 enzymes that are encoded by 27 different genes [1]. While a mutation in any of these genes may result in a leaf color mutant, most result in etiolated, albino or striped mutants, such as *CAO* [2] and *PORB* [3]. In most cases, mutations cause the differential expression of photosynthesis-related genes. Leaf color mutants of *Lagerstroemia indica* result from mutations that affect the synthesis of chlorophyll and the expression levels of photosynthesis-related genes [4]. Chloroplasts of higher plants possess the highly conserved light-harvesting chlorophyll protein complexes of PSI (LHCI) and PSII (LHCII), which are not only noncovalently bound to a series of pigments (chlorophyll a, chlorophyll b and lutein) [5], but are also necessary for the stacking of thylakoid membranes. In *Arabidopsis thaliana*, significant decreases in the light-harvesting chlorophyll protein complexes are known to result in phenotypes with chlorophyll deficiency [6].

Thus far, few studies have identified the functional genes responsible for the production of dark-green color in leaves, and the molecular mechanisms by which such genes regulate leaf color have remained unclear. Liu et al. [7] first discovered that a single base mutation in the CAB-conserved domain of *BrFC2* in Chinese cabbage leads to a darkening in leaf color. While FC2 catalyzes Proto IX to produce heme, an important regulator of tetrapyrrole metabolism, cyanobacterial FC consists of a catalytic domain and a Chl a/b-binding (CAB) domain, both of which are connected by a spacer [8]. 

In this study, nine inbred lines of Chinese cabbage with slight differences in leaf color were graded precisely based on their chlorophyll content and reflectance spectra to determined the photosynthetic capacity and actual photosynthetic rate. In addition, we analyzed the gene sequence and protein structure of dark-green leaf mutant gene *BrFC2* in inbred lines of Chinese cabbage to verify the universality of *BrFC2* mutation for dark-green leaf color. Additionally, we analyzed the relative expression of photosynthesis-related genes in the porphyrin and chlorophyll metabolism pathway as well as the photosynthesis pathway using qRT-PCR, aiming to reveal the differences in gene expression among different Chinese cabbage phenotypes with dark-green leaves, as well as the similarities in gene expression among phenotypes with the same leaf color. Our goal is to provide an empirical understanding of the candidate genes and molecular mechanisms responsible for the production of dark-green leaves in this economically important plant. 

## 2. Results

### 2.1. Phenotypic Identification of Chinese Cabbage Inbred Lines with Slight Differences in Leaf Color

**Figure 1:**
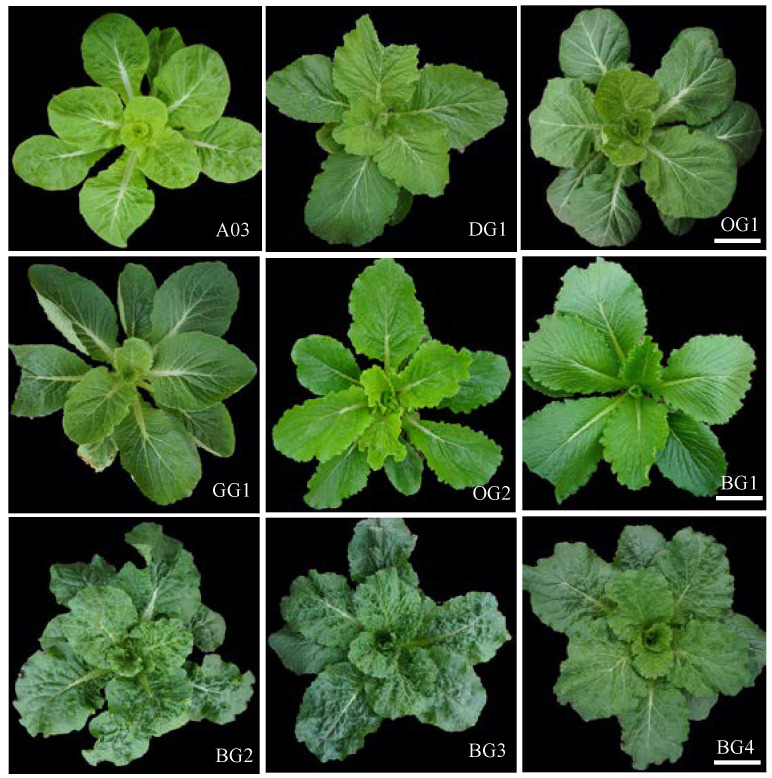
Phenotypic of Inbred Lines of Chinese Cabbage displaying Minor Variations in dark-green Leaves. Bar = 5 cm.

**Figure 2:**
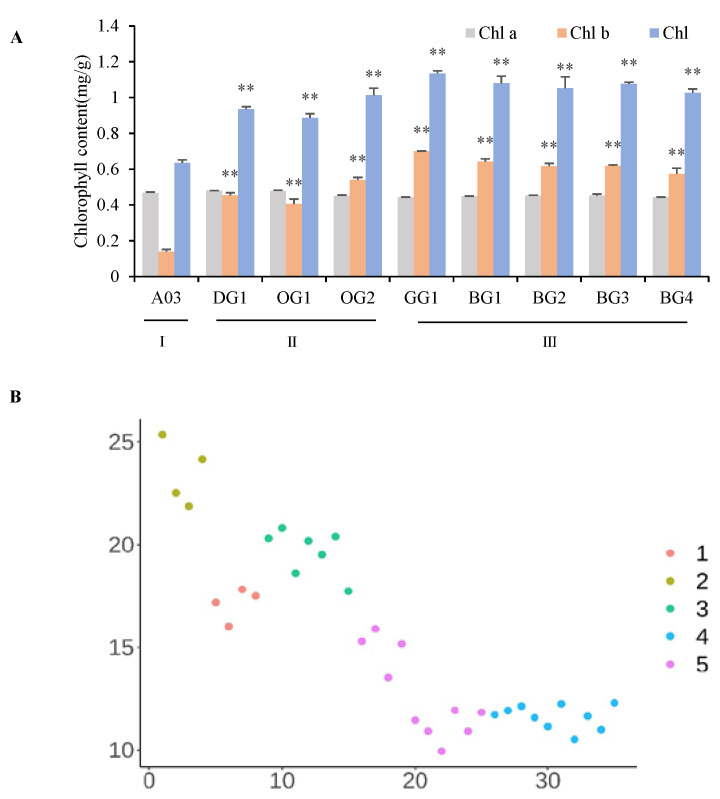
Leaf Color Classification of Inbred Lines of Chinese Cabbage displaying Minor Variations in dark-green Leaves. (**A**) Classifying the leaf color of Chinese cabbage inbred lines by chlorophyll content. (**B**) Cluster analysis of reflectance of nine inbred lines with slight differences in leaf color. ** *p* < 0.01.

Chlorophyll content was measured in nine inbred lines of Chinese cabbage with slight differences in leaf color (Figure 1), and we found that there was no significant difference in the content of chlorophyll a among the nine inbred lines of Chinese cabbage that displayed slight color differences. However, significant differences were observed in their content of chlorophyll b as well as their total chlorophyll content (Figure 2A). Based on their phenotypes and total chlorophyll content, we classified the nine inbred lines of Chinese cabbage into three classes: Class I, which contained the line A03 and had light-green leaves; Class II, which included the lines DG1, OG1 and OG2 and had oil-green leaves; and Class III, which included the lines GG1, BG1, BG2, BG3 and BG4 and had dark-green leaves.

To precisely grade the leaf color of the nine inbred lines of Chinese cabbage, the reflectance spectra were used to image each plant at different wavelengths to obtain spectral information on leaf color. The resultant spectral curves displayed the typical spectral characteristics of green vegetation, that is, darker colored leaves had lower reflectance (Figure S1). Cluster analysis was conducted on the reflectance of nine inbred lines with slight differences in leaf color at different wavelengths and were classified into five classes: Class I, including GG1 and BG1; Class II, including A03; Class III, including DG1; Class IV, including OG1 and OG2; and Class V, including BG2, BG3 and BG4.

### 2.2. Photosynthetic Parameters

**Figure 3:**
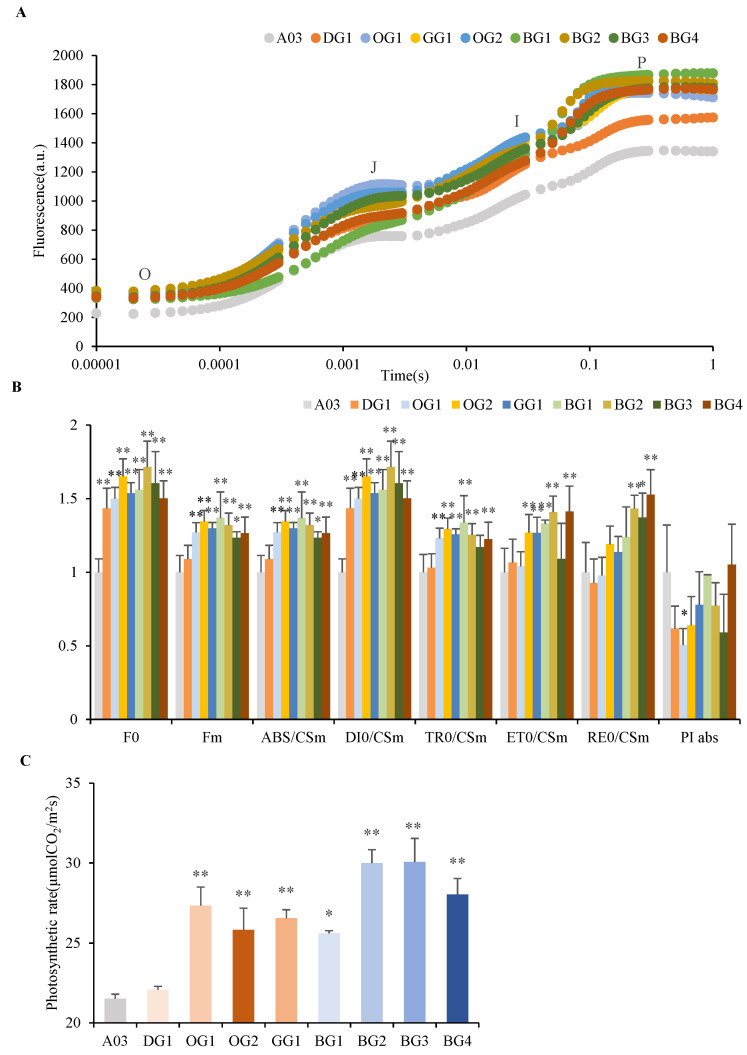
Photosynthetic Parameters of Inbred Lines of Chinese Cabbage displaying Minor Variations in dark-green Leaves. (**A**) OJIP transient curve of inbred lines of Chinese cabbage displaying minor variations in dark-green leaves. (**B**) JIP test parameter of inbred lines of Chinese cabbage displaying minor variations in dark-green leaves. (**C**) Photosynthetic rate of inbred lines of Chinese cabbage displaying minor variations in dark-green leaves. * *p* < 0.05, ** *p* < 0.01.

As the level of chlorophyll fluorescence can be indicative of the performance of photosynthetic devices, we analyzed differences in the level of chlorophyll fluorescence among the nine inbred lines of Chinese cabbage. The OJIP transient curve showed that the levels of chlorophyll fluorescence at O, J, I and P for eight lines with dark-green leaves were significantly higher than that of the line with light-green leaves (A03) (Figure 3A). The parameters of the JIP test were used to analyze the OJIP fluorescence transient curve. Here, the fluorescence parameter of line A03 (the line with light-green leaves) was set to 1. Figure 3B shows that the levels of initial fluorescence (F0) and maximum fluorescence (Fm) were significantly higher in the eight lines with dark-green leaves than in line A03, which had light-green leaves. Additionally, the levels of five specific fluxes—the absorption flux (ABS), dissipated energy flux (DI_0_), trapped energy flux (TR_0_), electron transport flux (ET_0_) and accepted electron flux (RE_0_)—were significantly higher in the eight lines with dark-green leaves on a per leaf cross-section (CSm) basis (Figure 3B). However, the five specific fluxes did not differ between the genotypes on a per reaction center (RC) basis (Figure S2). These results showed that the number of RCs per leaf cross-section (RC/CSm) in eight lines with dark-green leaves was significantly higher than that in line A03, which had light-green leaves. Photosynthetic Performance Index—PI abs has no significant difference in inbred lines, but significantly reduced in OG1.

To further validate these parameters, the photosynthetic rates of the nine inbred lines of Chinese cabbage were measured using a photosynthetic rate meter under a light intensity of 1000 μmol/m^2^s. We found that the eight lines with dark-green leaves had significantly higher photosynthetic rates than line A03, which had light-green leaves (Figure 3C). 

### 2.3. Gene Sequence and Structural Analysis of BrFC2

**Figure 4:**
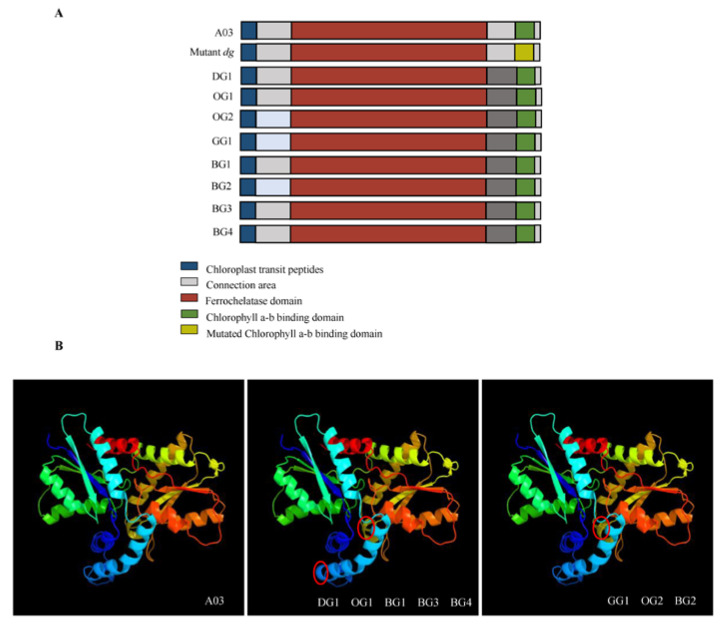
Gene sequence and protein structure of the *BrFC2* in Nine Inbred Lines of Chinese Cabbage displaying Minor Variations in Dark-green Leaves. (**A**) Sequence analysis of the *BrFC2* in nine inbred lines of Chinese cabbage displaying minor variations in dark-green leaves, (**B**) 3D structure prediction of three proteins.

We found that all nine inbred lines of Chinese cabbage that displayed slight differences in leaf color contained the ferrochelatase domain and the chlorophyll a-b binding (CAB) domains in *BrFC2*. Furthermore, the eight lines with dark-green leaves possessed the same conservative CAB domain found in line A03, which had light-green leaves; no single base mutation was observed in this domain (as described by Liu et al. [7]). In comparison with line A03, which had light-green leaves, the eight lines with dark-green leaves all possessed an additional glutamate (AGA) in the connecting region between the ferrochelatase domain and the CAB domain in BrFC2. The substitution of alanine with glycine was also found in the connecting region between the chloroplast transport peptide and the ferrochelatase domain in GG1, OG2 and BG2 (Figure 4A). 

The 3D protein structure of the above three types of proteins was predicted using the available web-based software PHYRE2. An additional glutamate in the connecting region between the ferrochelatase domain and the CAB domain resulted in an additional helix in the 3D protein structure of FC2 in corresponding lines such as DG1. This consequently tightened the other helix; the change in the two connecting regions would also lead to a tighter helix in corresponding lines, such as GG1 (Figure 4B). These results showed that the protein structure of BrFC2 was highly conserved in Chinese cabbage, and that any change in the amino acid sequence of this protein would alter its protein structure. At the same time, the molecular mechanisms responsible for the production of dark-green color in the eight lines of Chinese cabbage may have differed from those described by Liu et al. [7].

### 2.4. Expression Analysis of Photosynthesis-Related Genes

Liu et al. [7] analyzed the expression regulatory network in the dark-green leaf mutant dg using RNA-seq. To analyze the patterns of gene expression at the transcriptional level as well as any differences in photosynthesis-related genes, we analyzed the relative expression of photosynthesis-related genes involved in the chlorophyll metabolism pathway, photosynthesis pathways and photosynthesis-antenna protein pathways based on RNA-seq of Liu (Table S2).

**Figure 5:**
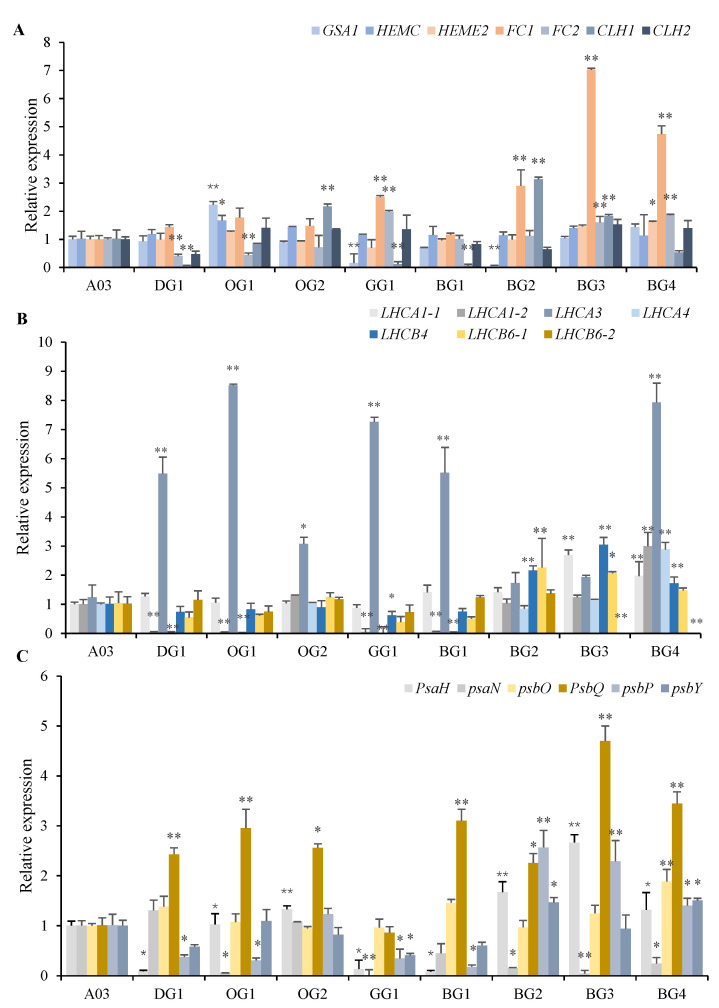
Expression Analysis of Photosynthesis-related Genes in Inbred Lines of Chinese Cabbage displaying Minor Variations in dark-green Leaves. (**A**) Gene relative expression of porphyrin and chlorophyll metabolism pathway. (**B**) Gene relative expression of photosynthesis-antenna proteins pathway. (**C**) Gene relative expression of photosynthetic pathway. * *p* < 0.05, ** *p* < 0.01.

In the porphyrin and chlorophyll metabolism pathway, *GSA1* catalyzes the production of ALA from Glu-tRNA. This is a rate-limiting step in the biosynthesis of tetrapyrrole and an important node in the regulation of chlorophyll synthesis. In comparison with the Chinese cabbage line A03, which had light-green leaves, we observed that the expression of *GSA1* in the oil-green Chinese cabbage inbred line OG1 was significantly higher, while that in the grayish-green Chinese cabbage inbred line GG1 and blackish-green Chinese cabbage inbred line BG2 was significantly lower. There was no differential expression of *GSA1* in the other lines whose leaves displayed different colors. *HEMC* catalyzes the production of uroporphyrinogen III (Uro III). We found that the expression of *HEMC* was significantly higher only in line OG1, which had oil-green leaves. We also observed that *HEME2* was only differentially expressed in line BG4, which had blackish-green leaves. We found that the expression of *FC1* was significantly increased in line GG1, which had grayish-green leaves, as well as in lines BG2, BG3 and BG4, which had blackish-green leaves. The expression of *FC2* was significantly decreased in line DG1, which had dark-green leaves, and OG1, which had oil-green leaves, and significantly increased in line GG1, which had grayish-green leaves, and lines BG3 and BG4, which had blackish-green leaves. We also found that the expression of *CLH1* was significantly decreased in DG1, GG1 and BG1, and significantly increased in OG2, BG2 and BG3. However, there are no significant differences in the expression of *CLH2* among the eight lines with dark-green leaves (Figure 5A). In summary, these results suggest that different inbred lines of Chinese cabbage with the same leaf color phenotype can possess different regulatory mechanisms for chlorophyll synthesis.

As the eight inbred lines of Chinese cabbage with dark-green leaves had significantly higher chlorophyll contents, we selected the LHCs in the photosynthesis-antenna protein pathway for an expression analysis. We found that the expression of the PS I light-harvesting chlorophyll protein complex genes *LHCA1*, *LHCA2* and *LHCA4* had decreased significantly in DG1, OG1, GG1, BG1 and BG4. Meanwhile, the expression of *LHCA3* had increased significantly in all the inbred lines of Chinese cabbage with dark-green leaves. We found that the expression of *LHCB4* was significantly increased in lines BG2, BG3 and BG4, which had blackish-green leaves. The expression of *LHCB6_1* had increased significantly in BG2 and BG3, and the expression of *LHCB6_2* had decreased significantly in BG3 and BG4 (Figure 5B). Overall, these results showed that gene expression of the light-harvesting chlorophyll protein complex had significantly increased in the lines BG2, BG3 and BG4, which had blackish-green leaves.

The photosynthetic system comprises two reaction centers, PS I and PS II, that work in series to carry out photochemical reactions. The performance of the photosynthetic system can be assessed based on an analysis of the expression of genes involved in the photosynthetic pathway. We found that the H subunit of PS I (*PsaH*) was differentially expressed in the eight inbred lines of Chinese cabbage which had dark-green leaves. Specifically, its expression was significantly decreased in line DG1, which had dark-green leaves, line GG1, which had grayish-green leaves, and line BG1, which had blackish-green leaves, but was significantly increased in lines OG1 and OG2, which had oil-green leaves, and lines BG2, BG3 and BG4, which had blackish-green leaves. The expression of *PsaN* was significantly decreased in lines OG1, GG1, BG2, BG3 and BG4, and the expression of the *PsbQ* subunit was significantly increased in all eight lines with dark-green leaves. The expression of the *PsbP* subunit was significantly increased only in blackish-green leaf inbred lines BG2, BG3 and BG4, but decreased significantly in other inbred lines. With the exception of *PsaN*, the expression of all genes involved in PS I and PS II were significantly increased in the blackish-green leaf inbred lines BG2, BG3 and BG4 (Figure 5C). This indicated that inbred lines of Chinese cabbage with darker leaf colors may have stronger photosynthetic structures.

**Figure 6:**
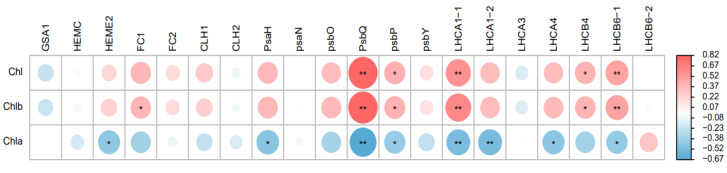
Correlation Analysis between Chlorophyll Content and Gene Relative Expression. * *p* < 0.05, ** *p* < 0.01.

Correlation analysis was conducted to better summarize the leaf color and expression levels of photosynthetic-related genes. The results showed that there was a highly significant positive correlation between *PsbQ*, *LHCA1_1*, *LHCB6_1* and chlorophyll b content, and a significant negative correlation between *PsbQ*, *LHCA1_1*, LHCA1_2 and chlorophyll a content (Figure 6).

## 3. Discussion

Phenotypic investigation is key to the selection of excellent varieties and the understanding of gene function. Research on plant phenotypes can promote the advancement of functional genomics and molecular methods for cultivating plant species. In this study, nine inbred lines of Chinese cabbage showing minor variations in leaf color were classified and graded. Specifically, based on their specific chlorophyll content, the nine inbred lines were classified into three categories: light-green, oil-green and dark-green. However, the coarse resolution of the grading system based on chlorophyll content meant that it could not further distinguish Chinese cabbage lines with only slight differences in leaf color. We used the reflectance spectra to further subdivide the nine inbred lines of Chinese cabbage into five categories. Image analysis (RGB, HSL) was also used to identify leaf color or fruit color [9]. The accurate identification of slight phenotypic differences in leaf color will provide an important empirical foundation for future efforts to locate candidate genes and to understand the molecular mechanisms determining leaf color phenotypes. The accuracy of the reflectance spectra in this study still requires further verification with image analysis.

In higher plants, Chlorophyll, including chlorophyll a and chlorophyll b, is the most abundant tetrapyrrole. Chlorophyll a is required for the formation of photosynthetic reaction centers and light-harvesting complexes; chlorophyll b acts as an auxiliary light capture pigment to help chlorophyll a carry out photosynthesis [10]. Chlorophyll b is also necessary for stabilizing the major light-harvesting chlorophyll-binding proteins [11]. At the same time, chlorophyll b can expand the spectral range of light absorbance of the LHCs, acclimate plant light regime and optimize the solar radiation utilization efficiency of (terrestrial) plants. Chlorophyll b-less barley, which contains fully functional reaction centers, has a relatively low photosynthetic capacity and greater sensitivity to high-intensity light because of a deficiency in LHCs [12]. Enhanced chlorophyll b synthesis has been reported to result in the accumulation of LHCs in transgenic Arabidopsis plants [13,14]. In this study, we found that the content of chlorophyll b in the eight lines with dark-green leaves was significantly higher than that of the A03 line which had light-green leaves, while it had no significant difference in chlorophyll a, resulting in a significant decrease in chlorophyll a/b ratio < 1. The chlorophyll a/b ratio decreased to 0.8 in *Arabidopsis* transgenic plants [13]. Liu et al. [7] found that in Chinese cabbage dark-green leaf mutant *dg*, the chlorophyll b content significantly increased to almost equal to the chlorophyll a content, leading to chlorophyll a/b∼1. In addition, there are no such reports in any green plants. Therefore, the physiological significance of the flexibility of the chlorophyll a/b ratio of LHCII in higher plants is an interesting area that warrants further investigation.

The chlorophyll fluorescence showed that the strength of fluorescence signal at O, J, I and P for eight lines with dark-green leaves was significantly higher than that of the line with light-green leaves, indicating higher surface concentration of chlorophylls in the light harvesting complexes of lines with dark-green leaves. The higher photosynthetic efficiency in the eight inbred lines of Chinese cabbage with dark-green leaves may be due to a significant increase in chlorophyll b content, leading to changes in the LHCs’ size, because antenna size is an important factor for the efficiency of photosynthesis. At the same time, the expression of LHCs significantly increased in dark-green lines, which may also be related to changes in the LHCs’ size.

Ferrochelatase 1 and 2 (*FC1* and *FC2*) are terminal enzymes of heme biosynthesis, but are not derived from different copies of the same gene. Rather, they play different roles in the distribution of heme across different plant tissues [15,16]. *FC1* is a housekeeper enzyme that synthesizes heme and provides it to the whole cell [16,17,18]. As its main function is to resist plant stress, it does not affect the PSII and the synthesis of chlorophyll [17,18,19]. *FC2* is mainly involved in the photosynthetic system, where it acts on the chloroplast cytochrome b6f complex and affects the assembly of PSII. Previously, Liu et al. [7] found that a single base mutation in the CAB-conserved domain of *BrFC2* in Chinese cabbage can result in a dark-green leaf phenotype. When we analyzed the gene sequence and protein structure of *BrFC2* in eight different dark-green Chinese cabbage inbred lines, we found no differences in their CAB domains and observed that all lines lacked the conservative base mutation described by Liu et al. [7]. Furthermore, the eight dark-green lines possessed an additional glutamate in the connecting region between the ferrochelatase domain and the CAB domain. This junction is crucial for the catalytic activity of FC2 [20], and also affects the 3D structure of proteins. The potential effects of sequence differences in this junction on the catalytic efficiency of BrFC2 enzymes in inbred lines of Chinese cabbage deserve further investigation.

Because the chlorophyll contents and photosynthetic rates of the eight dark-green lines were significantly higher than those of the light-green A03 line, we analyzed the transcriptional levels of the genes that were involved in porphyrin and chlorophyll metabolism, the photosynthesis-antenna protein and the photosynthesis pathway. The results showed that *PsbQ* and *PsbP* expression is positively correlated with chlorophyll b content and negatively correlated with chlorophyll a content. *PsbP* and *PsbQ* proteins are extrinsic subunits of the photosystem II in green plants [21]. Studies suggest that *PsbP* and *PsbQ* play an important role in the adjustment of photosynthetic light reactions to environmental changes, and also play an important role in defining the architecture of PSII-LHC II supercomplexes in higher plants [22,23]. The abundance of PSII-LHCII supercomplexes decreased in the mutants lacking *PsbQ* and/or *PsbR* [24]. LHCs play important roles in regulating photosynthesis in plants’ response to environmental stress. Minor antenna complex—*LHCB6* is related to the connection of LHCII trimers and the core of PSII in higher plants [25,26,27], and affected the interactions of PSII subunits and the electron transport rate in grana membranes, especially for limiting plastoquinone diffusion [28,29]. LHCB6-deficient plants showed a decrease in light-limited photosynthetic rate and growth, a disordered thylakoid arrangement, a decrease in the number of grana membranes and an increase in the number of starch granules [29,30]. In this study, *LHCB6* expression is positively correlated with chlorophyll b content. It seems reasonable that the chlorophyll b content in the inbred lines of Chinese cabbage with dark-green leaves changed the LHCs’ size, thus significantly increasing the gene expression of *LHCB6*, *PsbQ* and *PsbP*.

## 4. Materials and Methods

### 4.1. Plant Materials and Growth Conditions

Nine high-generation inbred lines of Chinese cabbage with different leaf colors were selected from the germplasm resource bank of the Hebei Province Key Laboratory for Vegetable Germplasm Innovation and Utilization and sown in seedling dishes in a greenhouse at the Hebei Agricultural University experimental farm in Baoding, China. After 30 days of growth, all plants were transplanted to soil and cultivated normally. At the seedling stage, the third leaf from the interior was harvested from three biological replicate plants of each line. The leaves were used for the measurement of chlorophyll content, multispectral analysis, chlorophyll fluorescence and the measurement of photosynthetic rate. All samples were flash frozen in liquid nitrogen and stored at −80 °C for RNA extraction and relative expression analysis.

### 4.2. Chlorophyll Measurement and Multispectral Analysis

Chlorophyll was extracted following Porra et al. [31] and measured at 663, 646 and 470 nm with a spectrophotometer (UV-1800; Shimadzu, Kyoto, Japan). A reflectance spectra imaging system (Videometer Lab 4, Videometer, Denmark) was used to collect visible light images and multispectral data of the Chinese cabbage leaves.

### 4.3. Measurements of Photosynthetic Parameters

The level of chlorophyll fluorescence of each line of Chinese cabbage was measured using a Handy-PEA fluorimeter Multifunctional Plant Efficiency Analyzer (Hansatech Instruments Ltd., Pentney, Norfolk, UK). Nine biological replicates were measured for each line of Chinese cabbage. Each plant was kept in the dark for at least 30 min and attached to a measuring clip. The level of chlorophyll fluorescence was measured within 0.01 ms to 2 s. A detailed analysis of the parameters of chlorophyll fluorescence was performed using the JIP test described by Strasser et al. [32]. 

The photosynthetic efficiency of each Chinese cabbage line was measured using an LI-6400XT portable photosynthesis system (LI-COR Biosciences, Lincoln, NE, USA).

### 4.4. RNA Extraction and Quantitative Real-Time PCR (qRT-PCR)

Plant total RNA was isolated using an RNA extraction kit (Promega, Madison, WI, USA), and first-strand cDNA was synthesized using EasyScript One-Step gDNA Removal and a cDNA Synthesis kit (Vazyme, Nanjing, China), with Anchored Oligo (dT)_18_ used as a primer according to the manufacturer’s instructions. All qRT-PCR specific primers (listed in Table S1) were designed in Primer Premier 3 software. qRT-PCR analysis was performed on a LightCycler 96 real-time PCR detection system (Roche, Basel, Switzerland) using SYBR dye. The 2^−∆∆CT^ method was used to calculate the relative expression levels of the target genes [33]. All reactions were performed with three biological and technical replicates.

### 4.5. Statistical Analysis

Data on physiological parameters such as chlorophyll content, chlorophyll fluorescence and photosynthetic rate were statistically analyzed and visualized using the software SPSS 22.0 and Microsoft Excel 2019. Student’s *t* test was performed to check for statistically significant differences. Two levels of statistical significance were determined; these are indicated in the figures as follows: *, *p* < 0.05; **, *p* < 0.01.

## 5. Conclusions

In this study, we precisely classified the inbred lines of Chinese cabbage with slight differences in leaves into five classes with a reflectance spectra system. The photosynthetic and electron transfer rates of the inbred lines which had dark-green leaves were significantly higher than those of the light-green inbred line A03. The eight lines with dark-green leaves possessed the same conservative CAB domain found in line A03, which had light-green leaves; no single base mutation was observed in this domain, but the eight lines with dark-green leaves all possessed an additional glutamate (AGA) in the connecting region between the ferrochelatase domain and the CAB domain, resulting in a change in the 3D protein structure. There is a highly significant positive correlation between expression of *PsbQ*, *LHCA1_1*, *LHCB6_1* and chlorophyll b content, and a significant negative correlation between expression of *PsbQ*, *LHCA1_1*, *LHCA1_2* and chlorophyll a content.

## Data Availability

The data presented in this study are available on request from the corresponding author.

## Supplementary Materials

The following supporting information can be downloaded at: https://www.mdpi.com/article/10.3390/plants12112124/s1, Figure S1: Reflectance of inbred lines of Chinese cabbage by reflectance spectra; Figure S2: JIP test parameter of inbred lines of Chinese cabbage displaying minor variations in dark-green leaves; Table S1: List of primers used for RT-qPCR analysis; Table S2: Gene annotation for qRT-PCR.
