# Supplementary material for: Leaf Color Classification and Expression Analysis of Photosynthesis-Related Genes in Inbred Lines of Chinese Cabbage Displaying Minor Variations in dark-green Leaves
Source: Plants (Basel). 2023 May 27;12(11):2124. doi: 10.3390/plants12112124 (PMC10255825; doi:10.3390/plants12112124)
Supplement: Supplementary file 1 [file plants-12-02124-s001.zip › plants-2383054-supplementary.pdf]

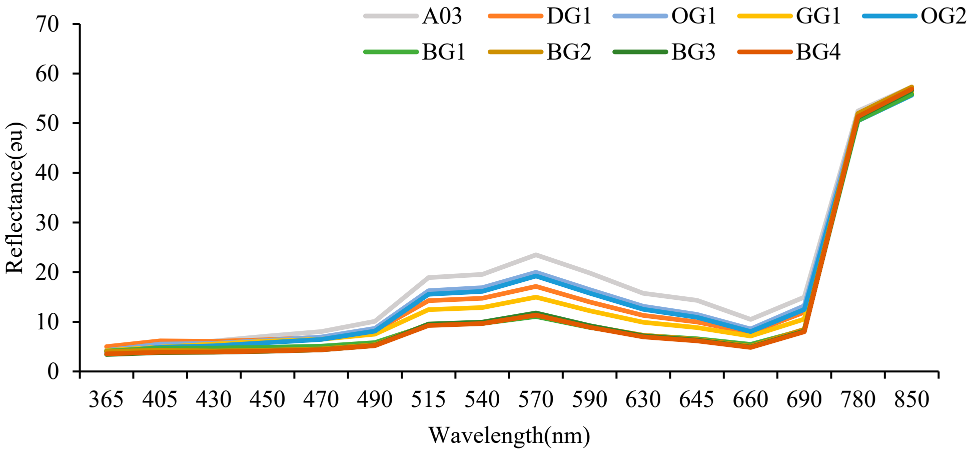

Supplementary Figure S1. Reflectance of inbred lines of Chinese cabbage by reflectance spectra

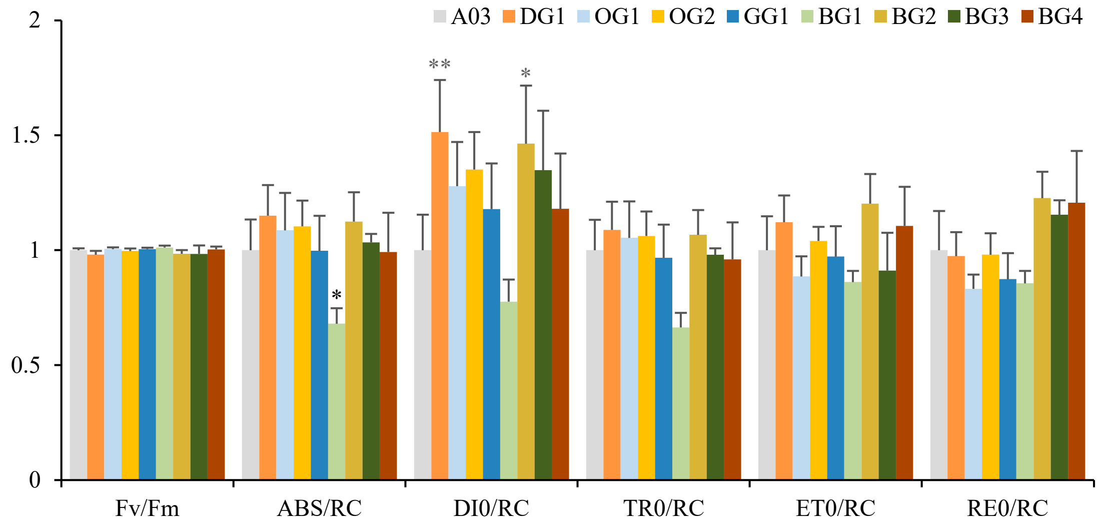

Supplementary Figure S2. JIP test parameter of inbred lines of Chinese cabbage displaying minor variation in dark-green leaf. * $p < 0.05$, ** $p < 0.01$.

Supplementary Table S1. List of primers used for RT-qPCR analysis

Gene	Gene ID	Forward primer sequence (5'-3')	Reverse primer sequence(5'-3')
GSA1	BraA06g027050.3C	GCTGGTAGTGGTGTAGCC	AGCCGTTAGAGTATCTGAAGTA
HEMC	BraA10g030600.3C	CAAGCCTACGAGACACGA	GAAGAGCCCTTTACCACC
HEME2	BraA05g001750.3C	GACTGGCTAGGACTGGTG	CCTTGAACCGTTATGTCTC
FC1	BraA06g033080.3C	TGTTGGGATGCGGTATTG	GCTTGAGCCAGTCGTGGA
FC2	BraA09g052590.3C	CGTTATTGGCATCCATTCACT	CGACTGTCGAGCTTCAAGGTTT
CLH1	BraA06g015220.3C	AGGCTACGAAGGGACATT	ACATACAACCCGCCATAA
CLH2	BraA09g019990.3C	CAGACACGATGGACGAGA	GTGCGAGTTTAGATAGGTTT
PsaN	BraA06g027510.3C	AGAGCATTCACCGTTCAG	TGTCTTTGCCTTCGCATT
PsaH	BraA06g002120.3C	CAACTGGTCAATGGGATC	TCCTCCTCCAAGAATCAA
PsbY	BraA07g033020.3C	GGCTCAAGGTAGCGACAA	CACCACCGATGCCAAGAC
PsbP	BraA10g005400.3C	CGAGACCCGTCCATTTAG	TCACCATAGGCAGCATCA
PsbO	BraA07g017380.3C	ACCGTGGCTCGTCTTTCT	TTCCTCGTCACCTCTACCTC
PsbQ	BraA09g024970.3C	GAACTTTCGGCGGTGATA	TCTCGTCCCTGTCTGCTC
LHCA1-1	BraA09g045720.3C	TCGGATACTCCAAAGACCC	AGCCTACAAACGCCAACA
LHCA1-2	BraA07g021900.3C	TACCCTTCCCTTCTTTCTT	ATCCAGTGAGCAGCCATT
LHCA3	BraA07g024640.3C	TATCTTCTTCATTTACCTCCTC	CCTGTTTGCTCCTTGCTT
LHCA4	BraA06g019540.3C	CTTAACTTCGCTCCTACGC	GGTCCCTTTCCAGTCACAT
LHCB4	BraA02g000180.3C	TAGCCAAGAACATAGCCG	GAGCCCAAAGACCTCACT
LHCB6-1	BraA06g011900.3C	TATGAGCCAGACAGGGAGAA	GGCACCTAGAGGCGTTTT
LHCB6-2	BraA09g058520.3C	TTTCGCAAACTACACTGGC	CCTATCTGGCTCATAAACACC
Bractin	BraA10g027990.3C	CGAAACAACTTACAACTCCA	CTCTTTGCTCATACGGTCA

Supplementary Table S2. Gene annotation for qRT-PCR

Pathway	Gene name	Gene ID	Homologys to Arabidopsis	Annotation
Porphyrin and chlorophyll metabolism	*GSA1*	BraA06g027050.3C	AT5G63570	Encodes glutamate-1-semialdehyde 2,1-aminomutase catalyzing the conversion of glutamate-1-semialdehyde (GSA) into 5-amino levulinate. The expression of this gene was demonstrated to be light-induced.
	HEMC	BraA10g030600.3C	AT5G08280	Encodes porphobilinogen deaminase.
	HEME2	BraA05g001750.3C	AT2G40490	Encodes uroporphyrinogen decarboxylase
	FC1	BraA06g033080.3C	AT5G26030	Encodes ferrochelatase I located in plastids. Involved in heme biosynthesis in non-photosynthetic tissues and induced by oxidative stress in photosynthetic tissues to supply heme for defensive hemoproteins
	FC2	BraA09g052590.3C	AT2G30390	Encodes one of two ferrochelatase genes in Arabidopsis. FC2 is speculated to operate in photosynthetic cytochromes.
	CLH1	BraA06g015220.3C	AT1G19670	Chlorophyllase is the first enzyme involved in chlorophyll degradation. It catalyzes the hydrolysis of the ester bond to yield chlorophyllide and phytol. Its expression is induced rapidly by methyljasmonate, a known promoter of senescence and chlorophyll degradation.
	CLH2	BraA09g019990.3C	AT5G43860	Chlorophyllase is the first enzyme involved in chlorophyll degradation. AtCLH2 has a typical signal sequence for the chloroplast. Gene expression does not respond to methyljasmonate.
Photosynthetic	*PsaN*	BraA06g027510.3C	AT5G64040	Encodes the only subunit of photosystem I located entirely in the thylakoid lumen.
	PsaH	BraA06g002120.3C	AT1G52230	Encodes the H subunit of photosystem I.
	PsbY	BraA07g033020.3C	AT1G67740	Encodes the Y subunit of photosystem Ⅱ. Important for the redox control of cytochrome b559.
	PsbO	BraA07g017380.3C	AT5G66570	Encodes the O subunit of photosystem II which is an extrinsic subunit of photosystem II and which has been proposed to play a central role in stabilization of the catalytic manganese cluster.
	PsbP	BraA10g005400.3C	AT1G06680	Encodes the O subunit of photosystem II and participates in the regulation of oxygen evolution.
	PsbQ	BraA09g024970.3C	AT4G05180	Encodes the Q subunit of the oxygen evolving complex of photosystem II.
Photosynthesis -antenna proteins	*LHCA1-1*	BraA09g045720.3C	AT3G54890	Encodes a component of the light harvesting complex associated with photosystem I.
	LHCA1-2	BraA07g021900.3C	AT3G54890	Encodes a component of the light harvesting complex associated with photosystem I.
	LHCA3	BraA07g024640.3C	AT3G61520	PSI type III chlorophyll a/b-binding protein
	LHCA4	BraA06g019540.3C	AT3G47470	Encodes a chlorophyll a/b-binding protein that is more similar to the PSI Cab proteins than the PSII cab proteins. The predicted protein is about 20 amino acids shorter than most known Cab proteins.
	LHCB4	BraA02g000180.3C	AT5G01530	Light harvesting chlorophyll-binding protein, CP29 is a component of the PSII light harvesting antenna complex.
	LHCB6-1	BraA06g011900.3C	AT1G15820	Lhcb6 protein (Lhcb6), light harvesting complex of photosystem II.
	LHCB6-2	BraA09g058520.3C	AT1G15820	Lhcb6 protein (Lhcb6), light harvesting complex of photosystem II.
